# Activity modulation and allosteric control of a scaffolded DNAzyme using a dynamic DNA nanostructure†
†Electronic supplementary information (ESI) available. See DOI: 10.1039/c5sc03705k


**DOI:** 10.1039/c5sc03705k

**Published:** 2015-10-26

**Authors:** Xiuhai Mao, Anna J. Simon, Hao Pei, Jiye Shi, Jiang Li, Qing Huang, Kevin W. Plaxco, Chunhai Fan

**Affiliations:** a Division of Physical Biology & Bioimaging Center , Shanghai Synchrotron Radiation Facility , CAS Key Laboratory of Interfacial Physics and Technology , Shanghai Institute of Applied Physics , Chinese Academy of Sciences , Shanghai , China . Email: fchh@sinap.ac.cn; b Department of Chemistry and Biomolecular Science and Engineering Program , University of California , Santa Barbara , California 93106 , USA; c Kellogg College , University of Oxford , Oxford , OX2 6PN , UK; d UCB Pharma , 208 Bath Road, Slough , SL1 3WE , UK . Email: jiye.shi@ucb.com; e School of Life Science and Technology , ShanghaiTech University , Shanghai 201200 , China

## Abstract

Recognition of the fundamental importance of allosteric regulation in biology dates back to not long after its discovery in the 1960s. Our ability to rationally engineer this potentially useful property into normally non-allosteric catalysts, however, remains limited. In response we report a DNA nanotechnology-enabled approach for introducing allostery into catalytic nucleic acids. Specifically, we have grafted one or two copies of a peroxidase-like DNAzyme, hemin-bound G-quadruplex (hemin-G), onto a DNA tetrahedral nanostructure in such a manner as to cause them to interact, modulating their catalytic activity. We achieve allosteric regulation of these catalysts by incorporating dynamically responsive oligonucleotides that respond to specific “effector” molecules (complementary oligonucleotides or small molecules), altering the spacing between the catalytic sites and thus regulating their activity. This designable approach thus enables subtle allosteric modulation in DNAzymes that is potentially of use for nanomedicine and nanomachines.

## Abstract 2

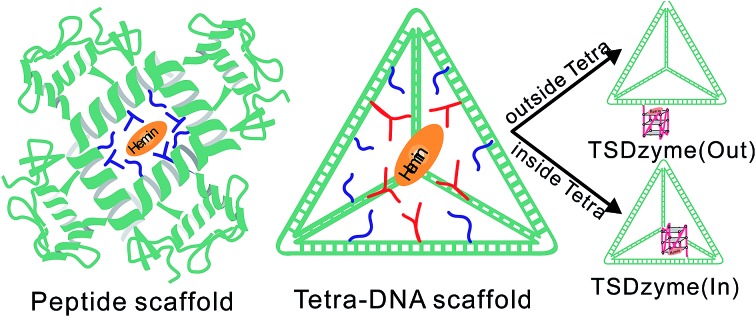
We report a DNA nanotechnology-enabled approach for the rational design of an allosteric deoxyribozyme by precisely and dynamically controlling the nanometer-scale interactions of two catalytic centers within a well-defined tetrahedral DNA scaffold.

## Introduction

Allosteric regulation, in which the binding of an effector at one site on a biomolecule regulates the function of a second, distal site,^1^,^2^ is used throughout biology to modulate such diverse cellular processes as catalysis, signal transduction and metabolism.^3^ Given its widespread use in nature, the rational introduction of allostery into designed biomolecules is expected to provide a flexible way to bring about improved control over function.^4^–^7^ Despite its potential value, however, there is a lack of a generic, easily realizable means of rationally engineering this property into normally non-allosteric biomolecules.^8^,^9^

**Fig. 1:**
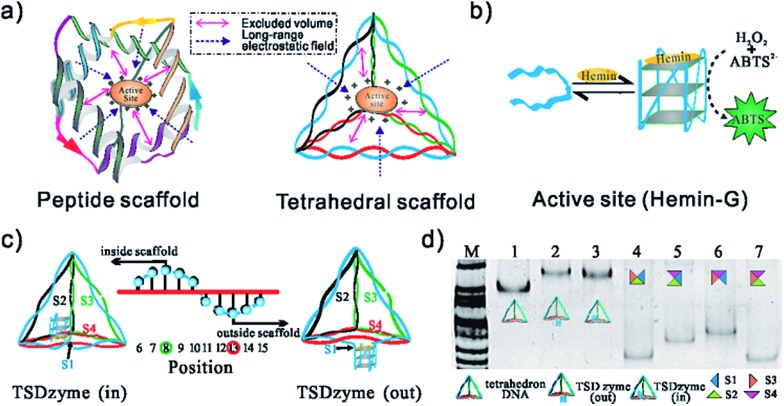
(a) DNA tetrahedral scaffolds provide a means of controlling the spacing and environment. (b) A schematic of the DNAzyme we have employed (hemin-G), and the colorimetric assay we used to monitor its activity. (c) Placement of the hemin-G can be either inside (TSDzyme (in)) or outside (TSDzyme (out)) of the tetrahedral DNA nanostructure which, as we show below, alters its catalytic properties. Here the black arrow indicates the hemin-G attachment site at the 5′ end of the first of the four strands that make up the tetrahedron (S1). The helical turn of the hemin attachment site determines the spatial placement of the catalytic domain. (d) The structures of the tetrahedral DNA nanostructures were characterized using native PAGE analysis. The lanes 1–3 are the bare DNA tetrahedron, TSDzyme (out) and TSDzyme (in), respectively. Lanes 4–7 are controls lacking one of the four strands required for the formation of the tetrahedron. Lane M contains DNA molecular weight markers.

DNA nanotechnology provides a powerful means to controllably organize biomolecules at the nanoscale,^10^–^12^ and thus may provide a route by which allostery can be rationally engineered into otherwise non-allosteric biocatalysts. Previous studies have proven, for example, that designed DNA nanostructures can span three dimensions and are capable of presenting biomolecules with tight control over spatial orientation.^13^–^21^ Motivated by this, we exploit here the organization ability of a tetrahedral DNA nanostructure to engineer a deoxyoligonucleotide (DNAzyme)/scaffold chimera exhibiting allosteric regulation. Specifically, by incorporating hemin-G, a G-quadruplex-based DNAzyme mimicking the activity of horseradish peroxidase, into a tetrahedral DNA nanostructure, we have created an easily optimized, higher-ordered structure (a tetrahedron-scaffold DNAzyme, or “TSDzyme”).^22^–^24^ The finely programmable structure of the tetrahedral nanostructure allows the local environment around hemin-G to be finely tuned, thus allowing in turn the introduction of tailorable allostery (Fig. 1).

## Results and discussion

### Single-active-site TSDzymes

To explore the extent to which attachment to our rigid, tetrahedral scaffold alters the physics of hemin-G, we first synthesized and characterized a TSDzyme consisting of a single catalytic DNAzyme, hemin-G, grafted onto a DNA tetrahedral scaffold (Fig. 1a and b). As the 7 nm sides of the tetrahedral scaffold are composed of double-stranded DNA and thus are not free to rotate, the position of each oligonucleotide in the helical strands and the position of the hemin-G on the tetrahedral scaffold are fixed. We can site-specifically position the ∼3 nm hemin-G either inside or outside the tetrahedral scaffold with near-Ångstrom precision, with the helical turn of the anchoring site determining placement (Fig. 1c).^25^

**Fig. 2:**
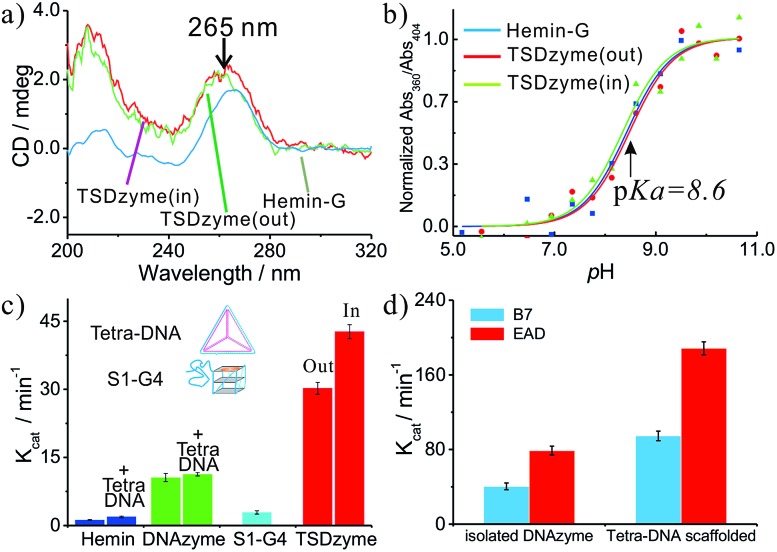
Structural and activity analysis of single-active-site TSDzymes. (a) CD spectra of free hemin-G, TSDzyme (out) and TSDzyme (in), all at 2.0 μM; the peak at ∼263 nm is characteristic of the formation of a parallel G-quadruplex, and indicates that the scaffold-bound hemin-G retains the parallel G-quadruplex conformation adopted by the isolated catalytic domain. (b) The p*K*_a_ values of free hemin-G, TSDzyme (out) and TSDzyme (in) are identical, suggesting that the degree of ionisation of a catalytically important water molecule in the catalytic domain is unchanged between the three. (c) Catalytic activity depends on the placement of the hemin-G active site. It is shown that *K*_cat_ of the free hemin-G (10.6 ± 0.9 min^–1^) increases 3-fold (to 30.2 ± 1.3 min^–1^) when the hemin-G is incorporated outside the tetrahedral scaffold; meanwhile, it increases 4-fold (to 42.7 ± 1.5 min^–1^) when it is incorporated inside the scaffold. (d) The tetrahedral scaffold also enhances the catalytic activity of other DNAzymes, such as EAD and B7.

Native polyacrylamide gel electrophoresis (PAGE) confirmed the formation of TSDzymes and the scaffold positioning of the hemin-G: “out” (*i.e.*, with the hemin-G positioned outside the tetrahedron) or “in” (hemin-G positioned inside), as described above (Fig. 1d). Grafting the hemin-G onto the tetrahedral scaffold does not induce major changes to either the structure or the mechanism of the catalytic domain. For example, the appearance of a specific spectral signature at ∼263 nm in circular dichroism (CD) confirms that the scaffold-bound G-quadruplex retains the parallel conformation adopted by the isolated quadruplex (Fig. 2a).^26^ Although we had suspected that the negative charge environment and restricted volume of the tetrahedral scaffold would stabilize the G-quadruplex structure through excluded-volume and long-range electrostatic effects, the folding free energy of the hemin-G placed on the inside of the scaffold is indistinguishable from that of hemin-G placed on the outside of the scaffold (Table S2,† –2.8 ± 0.6 kJ mol^–1^*vs.* –2.6 ± 0.9 kJ mol^–1^). We also examined the degree of ionisation (p*K*_a_) of a catalytically important water molecule in hemin-G, since hydrogen peroxide substrate must displace this water molecule in order to bind to the hemin center during the catalysis.^27^,^28^ Again, titration analysis revealed that this is effectively indistinguishable between free hemin-G and the scaffold-bound catalyst (Fig. 2b).

We next interrogated the effects of grafting on the catalytic activity of the hemin-G when attached to the scaffold. We measured this using a peroxidase-type colorimetric assay in which the hemin-produced hydrogen peroxide is used to convert a chromogenic substrate (2,2′′-azinobis[3-ethylbenzothiazoline-6-sulfonic acid]-diammonium salt, ABTS) into colored products. The Michaelis–Menten constants (*K*_M_) of free hemin-G and hemin-G on a tetrahedron (whether placed inside or outside the tetrahedron) are nearly identical (Table 1), suggesting that the affinity with which the catalyst binds its substrate is not altered. This result further confirms that the tetrahedral scaffold does not significantly change its catalytic mechanism. Grafting to the nanostructured scaffold, however, does enhance the catalytic activity of the DNAzyme. Specifically, *K*_cat_ of the free hemin-G (10.6 ± 0.9 min^–1^) increases 3-fold (to 30.2 ± 1.3 min^–1^) when the hemin-G is incorporated outside the tetrahedral scaffold. Also, *K*_cat_ increases 4-fold (to 42.7 ± 1.5 min^–1^) when it is incorporated inside the scaffold. A control study shows that direct incubation of either hemin-G or hemin with the DNA tetrahedron does not significantly alter the catalytic activity (Fig. 2c). In addition, when hemin-G is connected with a ssDNA S1, which forms one edge of the tetrahedral scaffold, the activity decreases by ∼70%, implying that the observed activity enhancement comes from the well-constructed tetrahedral scaffold.

**Table 1:** Kinetics, dissociation constants, and *K*_cat_ for free hemin, DNAzyme, TSDzyme (out), TSDzyme (in), and allosteric TSDzyme

	*K*_M_ (mM)	*K*_cat_ (min^–1^)	*K*_D_ (μM)
Hemin	14.7 ± 1.2	1.2 ± 0.1	—
Free hemin-G	2.51 ± 0.17	10.6 ± 0.9	2.2 ± 0.5
TSDzyme (out)	2.21 ± 0.14	30.2 ± 1.2	0.75 ± 0.23
TSDzyme (in)	2.30 ± 0.14	42.7 ± 1.5	0.70 ± 0.14
Allosteric TSDzyme	2.19 ± 0.20	—	0.45 ± 0.17

The observed increase in catalytic activity appears to arise due to improved affinity of the G-quadruplex for hemin (*i.e.* decreased *K*_D_). Specifically, the *K*_D_ values of the two TSDzymes (0.75 ± 0.23 μM and 0.70 ± 0.14 μM for the out and in configurations, respectively) are 3-fold higher than that of the isolated hemin-G (2.2 ± 0.5 μM) (Fig. S1† and Table 1). This increase in the binding affinity arises from the stabilization of hemin-G in the presence of the tetrahedral scaffold.^29^–^31^ In addition to this effect, the presence of the bulky tetrahedral scaffold may prevent oligomerization of the hemin-G, which can decrease binding affinity and catalytic activity (Fig. S2†).^32^–^34^

**Fig. 3:**
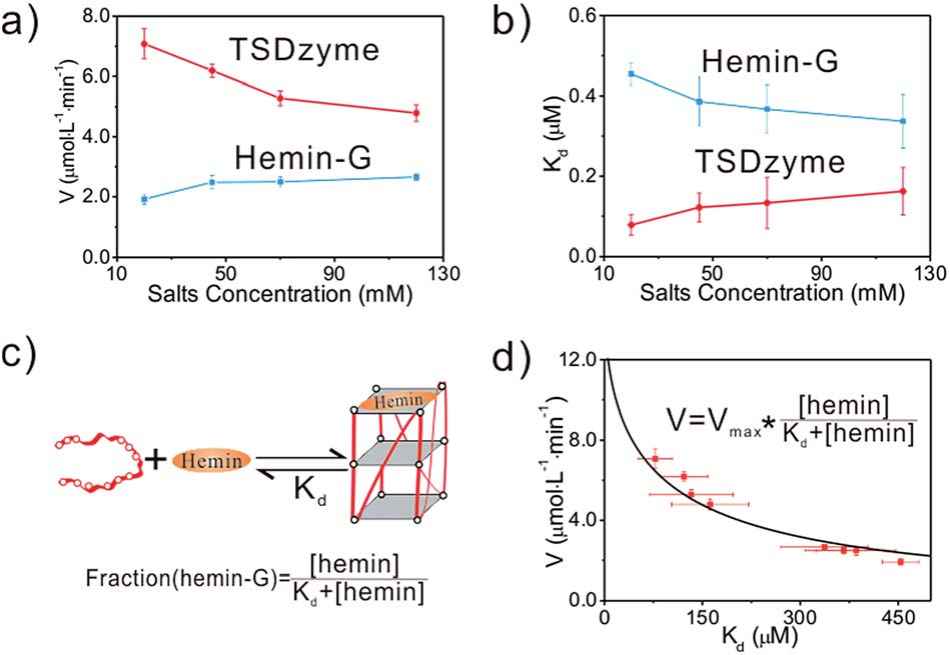
(a and b) Quantification of the catalytic activity and dissociation constant as a function of increased bulk salt concentration. (c and d) A schematic of the DNAzyme we have employed and simulation of the relationship between the catalytic activity and dissociation constant of the catalytic group for hemin, which suggests that the observed increase in catalytic activity arises due to an increase in the affinity of the G-quadruplex for hemin. The simulation follows the equation: *K*_cat_ = *K*maxcat × [hemin-G]/([hemin-G] + [G-quadruplex]) = *K*maxcat × [hemin]/([hemin] + *K*_D_).

Since the DNA tetrahedron is composed of six polyanionic DNA strands, this negatively charged environment should lead to an electrostatic effect on the DNAzyme. To test this, we increased the ionic strength of the solution, which resulted in a decrease in the activity of the TSDzyme and an increase in *K*_D_ (Fig. 3a and b). We also note that the activity and *K*_D_ are negatively correlated (Fig. 3d).

To demonstrate the versatility of this tetrahedral scaffold platform, we chose other types of G-quadruplex with a similar parallel structure, including EAD and B7.^35^,^36^ Similarly to the G-quadruplex-based TSDzyme, the catalytic efficiencies of both EAD and B7 are increased 2-fold when they are incorporated into the tetrahedral scaffold, which further substantiates that the observed activity enhancement comes primarily from the tetrahedral scaffold (Fig. 2d).

### Bivalent, allosteric TSDzymes

**Fig. 4:**
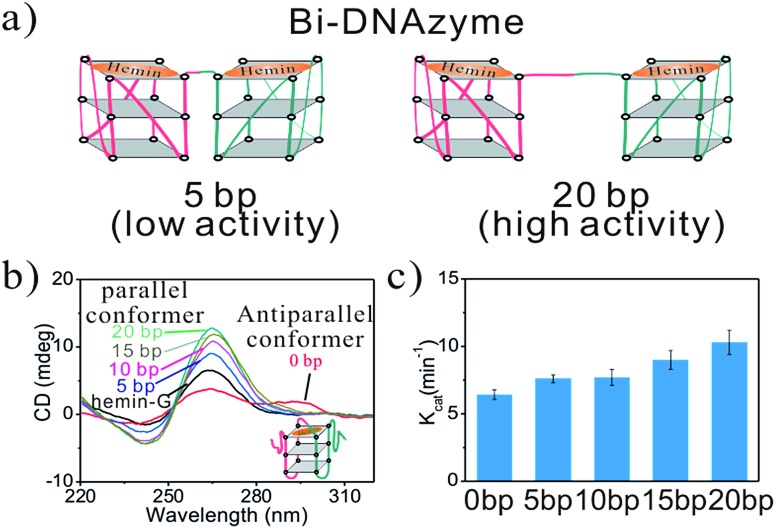
The activity of the hemin-Gs in bivalent TSDzyme constructs is dependent on their spacing. (a) Schematic of the bi-hemin-Gs we inserted into TSDzymes. (b) The CD spectra of these confirm that they retain the parallel G-quadruplex conformation of the free G-quadruplex. (c) The catalytic activity of the bi-hemin-Gs increases with increasing spacer length, as shown by the generated *K*_cat_ values of various bi-hemin-Gs.

The functionality of the hemin-G in single-active-site TSDzyme motivated us to design multivalent TSDzymes supporting allosteric control (allosteric TSDzymes). To do so, we designed bivalent TSDzymes in which the two hemin-Gs are placed together on one edge of the tetrahedral scaffold. Our rationale is that if two catalytic groups are positioned too closely together, they will mutually reduce the local substrate concentration, impeding catalysis.^37^ To study this effect, we designed a series of scaffold-free bi-hemin-Gs that contain two hemin-Gs linked by oligonucleotide spacers with lengths of 0 bp, 5 bp, 10 bp, 15 bp and 20 bp (Fig. 4a), and tested whether adjacent hemin-Gs interact with each other, altering their catalytic activity. The CD data demonstrate that the adjacent hemin-Gs in each of the bi-hemin-Gs remain structurally similar to the parallel conformation of free hemin-G, although bi-DNAzyme without the spacer adopts an intermolecular anti-parallel structure (Fig. 4b). Consistent with this, the hemin *K*_D_ values of the various bi-hemin-Gs are nearly identical (Table S3†). The catalytic activity of the bivalent TSDzymes, however, depends strongly on the relative geometry of the two catalytic groups, with *K*_cat_ inversely dependent on the distance between the two hemin-Gs (Fig. 4c). For example, the bi-hemin-G with the greatest distance (20 bp) between adjacent hemin-Gs exhibits the highest *K*_cat_.

**Fig. 5:**
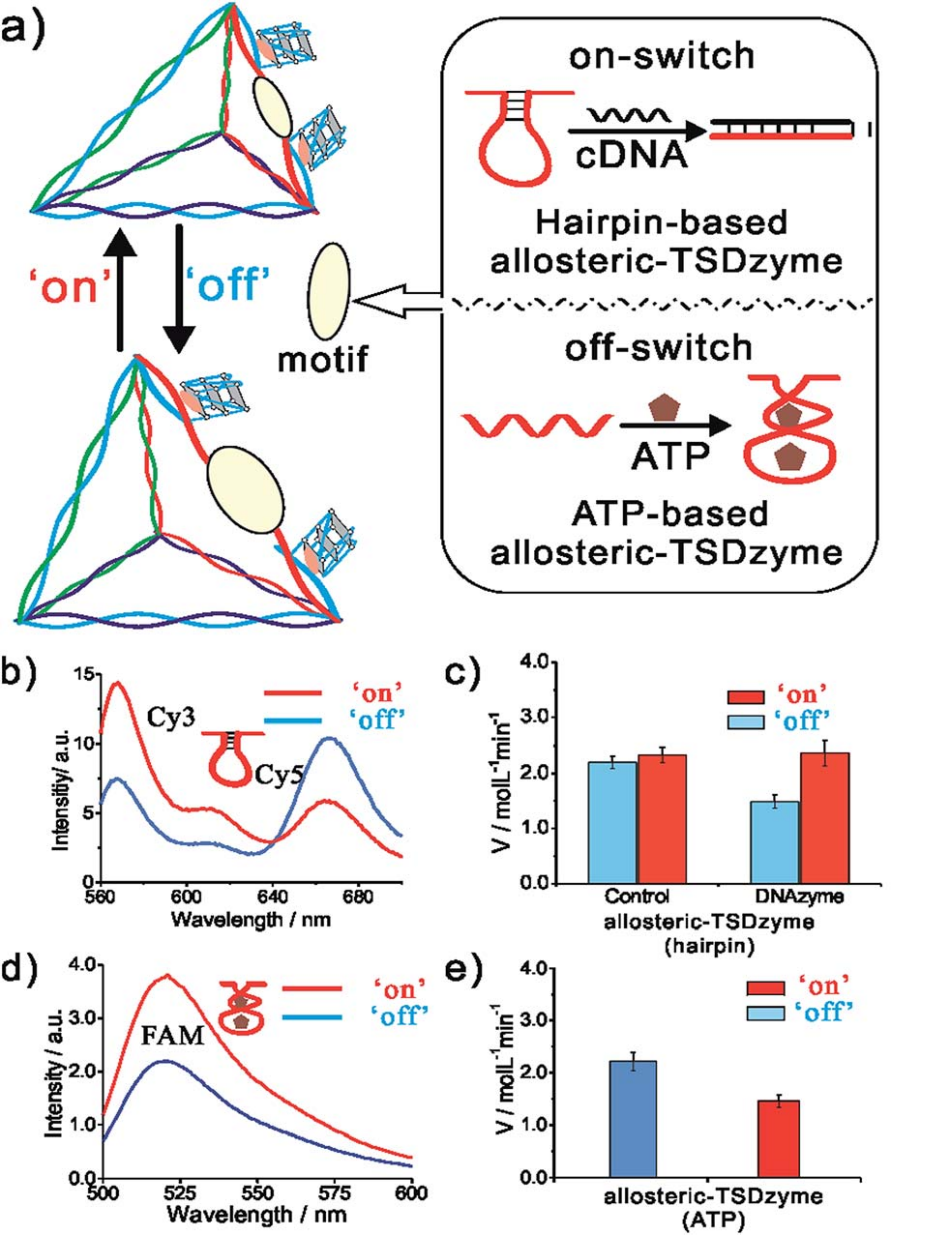
A DNA strand complementary to a reconfigurable hairpin motif in an allosteric TSDzyme serves as a biomolecular effector, allosterically modulating its catalytic activity. (a) Schematics of the hairpin-based and ATP-based allosteric TSDzyme. (b and d) FRET analysis confirms that the TSDzyme undergoes the expected structural reconfiguration in response to the binding of the DNA effector (200 nM). To see this, the DNA tetrahedron was labelled with either Cy3 donor and Cy5 acceptor or FAM and Dabcyl quencher in place of hemin-Gs on the reconfigurable edge, and thus variations in the ratio of donor to acceptor fluorescence indicate the length of this edge. (c and e) As expected, the catalytic activities of the allosteric TSDzymes are modulated by the presence of either the cDNA or ATP effector (0.5 mM), as shown by the generated *V* values for the allosteric TSDzyme.

The geometry-dependent activity of the hemin-Gs in the bivalent TSDzyme constructs provides a route by which we can employ the scaffold to introduce allosteric control. To modulate the distance between the adjacent hemin-Gs, and thus control their activity, we engineered a reconfigurable hairpin motif into the scaffold (Fig. 5a). The conformation of this motif, and thus the spacing between the catalytic groups, can be controlled by introducing a target cDNA that is complementary to the hairpin motif. Upon binding, this effector increases the distance between hemin-Gs from 1.7 nm to 6.8 nm, which is the equivalent of a change from a 5 base pair spacing to 20 base pairs. Native PAGE and fluorescence resonance energy transfer (FRET) confirm the change in the configuration of the tetrahedral scaffold and the change in the position of the adjacent hemin-Gs upon effector binding (Fig. 5b and S3†). Meanwhile, the CD data demonstrate that adjacent hemin-Gs within the tetrahedral scaffold adopt the same parallel structure as the free hemin-G (Fig. S4†). This hairpin-based, allosteric TSDzyme showed a similar response to the (non-allosteric) bi-hemin-Gs with regard to the relationship between inter-catalyst distance and catalytic activity. Specifically, binding of the cDNA effector increases catalytic efficiency by approximately 60% (from 1.48 ± 0.12 μM min^–1^ to 2.36 ± 0.24 μM min^–1^). Also of note is that a control tetrahedral scaffold without the hairpin motif does not show a significant change in *K*_M_ or the catalytic activity (Fig. 5c). We also tested the allosteric modulation using tetrahedral scaffolds incorporating EAD and B7, which showed a similar effect upon effector binding, demonstrating the versatility of this allosteric control (Fig. S5†).

While convenient as a proof-of-principle, the use of DNA as an allosteric regulator is perhaps of less value than the use of small molecule effectors (which would enable, for example, feed-back and feed-forward control of catalytic pathways in cells). To demonstrate the feasibility of this we next constructed an allosteric TSDzyme responsive to ATP. To do so, we engineered an ATP-binding aptamer sequence into one of the edges (Fig. 3a). Upon binding to ATP, the motif contracts, reducing the distance between the adjacent hemin-Gs (Fig. 5d and S6†). Consistent with this, *K*_cat_ of the allosteric TSDzyme decreases by approximately 50% upon ATP-binding, from 2.21 ± 0.18 μM min^–1^ to 1.45 ± 0.12 μM min^–1^ (Fig. 5e).

## Conclusions

Herein we have described a DNA nanotechnology-enabled approach for the redesign of DNAzymes with tailorable allostery. We have demonstrated that catalytic hemin-G can be grafted to a three-dimensional tetrahedral DNA scaffold. Significantly, bivalent TSDzymes with two active sites exhibit the reconfigurable flexibility required to generate allosteric regulation. More specifically, we have demonstrated effective allosteric regulation with either oligonucleotides or small molecules. Since a wide range of DNA nanostructures can be designed with high precision and synthesized at low cost, this approach compares favourably with protein engineering approaches. For example, previous efforts in manipulating protein structure rely on heavy computational power and are hampered by our still limited knowledge regarding protein folding.^38^–^41^ Especially, little attention has been paid to bringing about subtler modulation mechanisms, such as allosteric control, in such designed proteins. Hence, the demonstrated ability to regulate the catalytic efficiency of a biocatalyst opens new opportunities for designing biocatalysts for applications ranging from medical diagnostics and targeted therapeutics to bio-energy conversion.^42^,^43^

## Supplementary Material

Supplementary information
