# Supplementary material for: Activity modulation and allosteric control of a scaffolded DNAzyme using a dynamic DNA nanostructure
Source: Chem Sci. 2015 Oct 26;7(2):1200–4. doi: 10.1039/c5sc03705k (PMC5975834; doi:10.1039/c5sc03705k)
Supplement: Supplementary file 1 [file SC-007-C5SC03705K-s001.pdf]

Electronic Supplementary Material (ESI) for Chemical Science.
This journal is © The Royal Society of Chemistry 2015

Activity modulation and allosteric control of a scaffolded DNAzyme using a dynamic DNA nanostructure

Xiuhai Mao[a], Anna J. Simon[b], Jiye Shi[c,d*], Jiang Li[a], Qing Huang[a], Kevin W. Plaxco[b], Chunhai Fan[a*]

[a]Division of Physical Biology & Bioimaging Center, Shanghai Synchrotron Radiation Facility, CAS Key Laboratory of Interfacial Physics and Technology, Shanghai Institute of Applied Physics, Chinese Academy of Sciences, Shanghai, China

[b]Department of Chemistry and Biomolecular Science and Engineering Program, University of California, Santa Barbara, California 93106, United States

[c]Kellogg College, University of Oxford, Banbury Road, Oxford, OX2 6PN, UK

[d]UCB Pharma, 208 Bath Road, Slough SL1 3WE, United Kingdom

Experiments section:

Materials

Tris-(hydroxymethyl) aminomethane was from Cxbio Biotechnology Ltd. 2,2'-Azino-bis(3-ethylbenzthiazoline-6-sulfonic acid) diammonium salt (ABTS) and chloro [3,7,12,17-tetramethyl-8-13-divinylporphyrin 2,18-dipropanoato(2-)]iron(III) (hemin) were purchased from Sigma-Aldrich (St. Louis, MO). ATP was from Sangon Inc. (Shanghai, China). All solutions were prepared with Milli-Q water (18MΩ·cm resistivity) from a Millipore system. All oligonucleotides were synthesised and purified by Sangon Inc. Sequences are shown in Table S1.

Table S1. Oligonucleotides used in this work

Oligo	Sequence
S1	5'-CAG TTGAG ACGAA CATTC CTAAG TCTGA AATTT ATCAC CCGCC ATAGT AGACG TATCA CCAGG-3'
G-quadruplex	5'-GGGTA GGGCG GGTTG GG-3'
S1-outside tetrahedron DNA	5'-GGGTA GGGCG GGTTG GGCAG TTGAG ACGAA CATTC CTAAG TCTGA AATTT ATCAC CCGCC ATAGT AGACG TATCA CCAGG-3'
S1-inside tetrahedron DNA	5'-GGGTA GGGCG GGTTG GGCCA GGCAG TTGAG ACGAA CATTC CTAAG TCTGA AATTT ATCAC CCGCC ATAGT AGACG TATCA-3'
EAD	5'-CTGGG AGGGA GGGAG GGA-3'
B7	5'-ATTGG GAGGG ATTGG GTGGG-3'
EAD-S1	5'-CAG TTGAG ACGAA CATTC CTAAG TCTGA AATTT ATCAC CCGCC ATAGT AGACG TATCA CCAGG-3'
B7-S1	5'-CAG TTGAG ACGAA CATTC CTAAG TCTGA AATTT ATCAC CCGCC ATAGT AGACG TATCA CCAGG-3'
S2	5'-CTTGC TACAC GATTC AGACT TAGGA ATGTT CGACA TGCGA GGGTC CAATA CCGAC GATTA CAG

S3	5'-GGTGA TAAAA CGTGT AGCAA GCTGT AATCG ACGGG AAGAG CATGC CCATC CACTA CTATG GCG-3'
S4	5'-CCTCG CATGA CTCAA CTGCC TGGTG ATACG AGGAT GGGCA TGCTC TTCCC GACGG TATTG GAC-3'
Tetra 1	5'-AGGCA GTTGA GACGA ACATT CCTAA GTCTG AAATT TATCA CCCGC CATAG TAGAC GTATC ACC-3'
Tetra 2	5'-CTTGC TACAC GATTC AGACT TAGGA ATGTT CGACA TGCGA GGGTC CAATA CCGAC GATTA CAG-3'
Tetra 3-hairpin	5'-GGTGA TAAAA CGTGT AGCAA GCTGT AATCG ACTCT A**GCGG AAGAA CCCAC AACCG C**GGCT CACTA CTATG GCG-3'
Tetra 4	5'-GGGTA GGGCG GGTTG GGTTA GAGAC GGTAT TGGAC CCTCG CATGA CTCAA CTGCC TGGTG ATACG AGAGC CTGGG TAGGG CGGGT TGGG-3'
DNA	5'-GCGGT TGTGG GTTCT TCCGC CTTCC TCTCG-3'
Tetra 3-ATP	5'-GGTGA TAAAA CGTGT AGCAA GCTGT AATCG ACTCT A**ACCT GGGGG AGTAT TGCGG AGGAA GGT**GG CTCAC TACTA TGGCG-3'
Anti-ATP	5'-ACCTT CCTCC GCAAT ACTCC CCCAG GT-3'
5 bp Bi-quadruplex	5'-GGGTA GGGCG GGTTG GG **TTTTT** GGG TAGGG CGGGT TGGG -3'
10 bp Bi-quadruplex	5'-GGGTA GGGCG GGTTG GG **TTTTT TTTTT** GGG TAGGG CGGGT TGGG -3'
15 bp Bi-quadruplex	5'-GGGTA GGGCG GGTTG GG **TTTTT TTTTT TTTTT** GGG TAGGG CGGGT TGGG -3'
20 bp Bi-quadruplex	5'-GGGTA GGGCG GGTTG GG **TTTTT TTTTT TTTTT TTTTT** GGG TAGGG CGGGT TGGG -3'

Assembly of the tetrahedron scaffolds

Stoichiometric mixtures of component oligonucleotides were combined in TSD assembly buffer (10 mM Tris, 50 mM KCl, and 15 mM $MgCl_2$, pH 8.0), heated at 95°C for 5 min and then cooled at 4°C for 30 s with a PCR machine (Applied Biosystems, US).

Colorimetric Measurement Assay

This experiment was performed in a solution consisting of DNA (100 nM) and hemin (DNA: Hemin ratio 1:2), H_2O_2 (0.2~5 mM), ABTS (5 mM) in buffer (10 mM Tris-HCl, 50 mM KCl, and 15 mM $MgCl_2$, pH 8.0). DNA and Hemin were incubated at 37°C for 30 min prior to the addition of H_2O_2 and ABTS. To characterize the rate of oxidation of ABTS, absorbance changes at 420 nm were monitored by a microplate reader (TECAN Genios Pro, Switzerland). Based on the Michaelis equation, the kinetic parameters were calculated from V_{obs} at varying concentrations of H_2O_2 (0.2 mM, 0.4 mM, 0.6 mM, 0.8 mM, 1.0 mM, 1.5 mM, 2.0 mM, 2.5 mM, 3.0 mM, 3.5 mM and 4.0 mM respectively). The initial reaction rate V_{obs} was calculated from the changes in absorbance during the first 20 s. ε is 36000 $M^{-1} \cdot cm^{-1}$.

In the Michaelis-Menten kinetics, $V = K_{cat}*([Enzyme])*\ ([S] / ([S] + K_M))$, in which K_{cat} is the turnover number (the maximum number of substrate molecules converted to product per enzyme molecule per second) and K_M is the Michaelis constant (the substrate concentration at which the reaction rate is at half-maximum and an inverse measure of the substrate's affinity for enzyme).

Gel Electrophoresis

DNA samples were analyzed by 10% Native PAGE (29:1 Acrylamide: bisacrylamide mixture) using 1× TBE buffer and stained with Sybr Gold (Invitrogen, US).

Calculation of Binding Constants

The saturation curve for hemin-G formation was determined by plotting the absorbance changes at 404nm as a function of DNA concentration. The dissociation constant (K_d) was obtained by fitting the saturation plot according to the following equation.

$$[DNA]_0 = K_d\ (A-A_0) / (A_\infty-A) + [P_0]\ (A-A_0) / (A_\infty-A_0)$$

Where $[DNA]_0$ is the initial concentration of DNA; $[P_0]$ is the initial concentration of monomeric hemin; A_∞ and A_0 represent hemin absorbance at saturating DNA concentrations and in the absence

pKa Determination

The buffers used were PB buffer (pH 5.5-7.4), Tris-HCl buffer (pH 7.7-9.5) and Glycine-NaOH (pH 9.8-10.6). All buffer solutions contained 50 mM KCl to ensure proper DNA folding and the reactions were carried out at room temperature.

Circular Dichroism Experiments

Oligonucleotides were diluted to 5 μM in potassium acetate (KAc) buffer (20 mM KAc, 70 mM KCl, pH 6.8). The spectral was obtained with a Chirascan CD Spectrometer (Applied Photophysics Ltd, UK). The wavelength was varied from 200 nm to 320 nm at 100 nm min^{-1}. The samples were measured at 20°C with a square quartz cell with a 0.1 cm path length. The buffer spectrum was subtracted from each sample spectrum and smoothed.

Thermal Denaturation. Thermal denaturation experiments were carried out on a spectrophotometer (Carry), using 1-cm path length quartz cells. Oligonucleotides final concentration was 5 μM in potassium acetate (KAc) buffer (20 mM KAc, 70 mM KCl, pH 6.8). Absorbance was monitored as a function of the temperature at 295 nm for the determination of the melting temperature (Tm) and at 405 nm as control wavelength. Gradient was 0.3 °C/min between 40 °C and 90 °C.

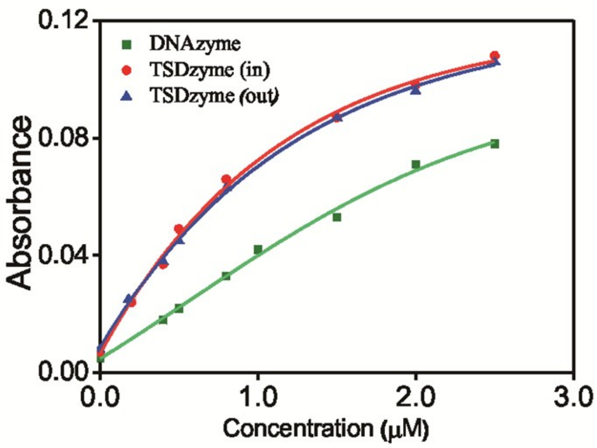

Figure S1. The dissociation constants (Kd) of hemin-G, TSD (out) and TSD (in) were determined by plotting the absorbance changes of hemin at 404 nm against the DNA concentration.

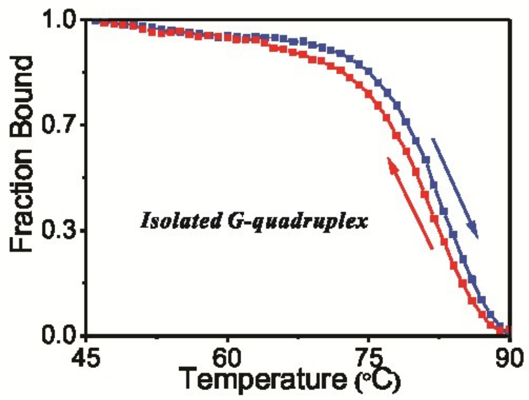

Figure S2. Thermal denaturation curves were recorded by monitoring absorbance at 295 nm in KAc buffer.

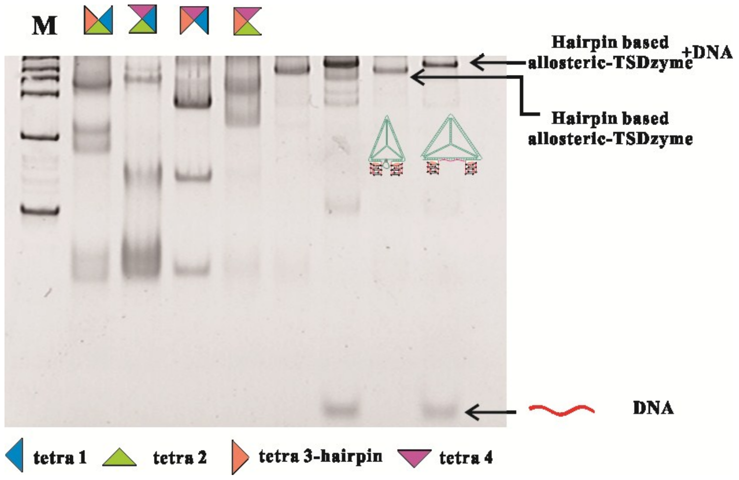

Figure S3. 10 % native gel electrophoresis of hairpin-based allosteric-TSDzyme formation. Lane 5 &7 stands for allosteric-TSDzyme with different concentration of 250 nM &100 nM, absent of cDNA. Lane 6 &8 stands for allosteric-TSDzyme with 500 nM cDNA. Lanes 1, 2, 3 & 4 stand for any other combination lacking one oligonucleotide as control. Lane M, 50 bp marker.

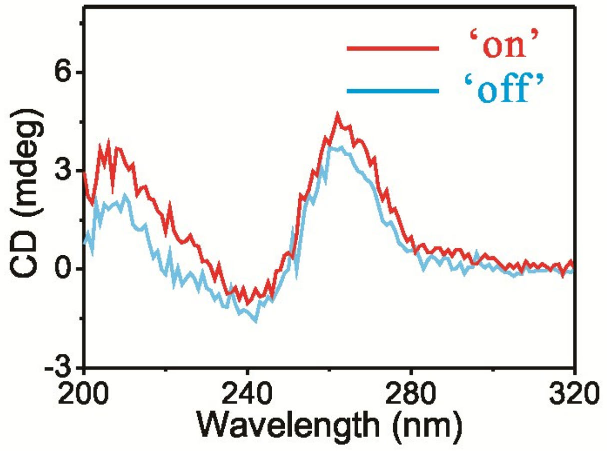

Figure S4. CD spectra of allosteric TSDzyme, all at 2.0 μM; the peak at ~263 nm is characteristic of the formation of a parallel G-quadruplex, and indicates that these adjacent DNAzymes incorporated with allosteric scaffold retain the parallel G-quadruplex conformation adopted by the isolated catalytic domain.

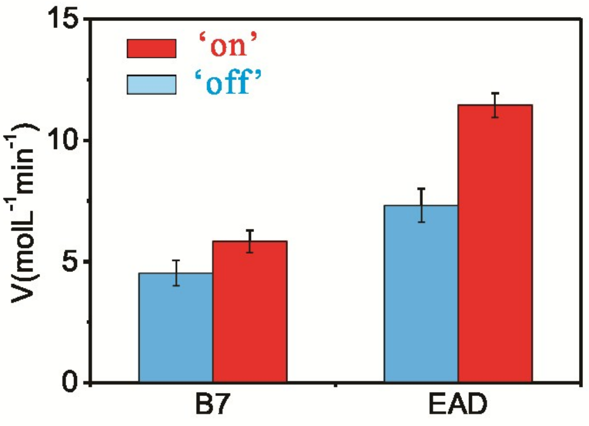

Figure S5. After adding cDNA, the catalytic activity of allosteric TSDzyme (hairpin) for B7 and EAD significantly increased for 30% and 50%, separately.

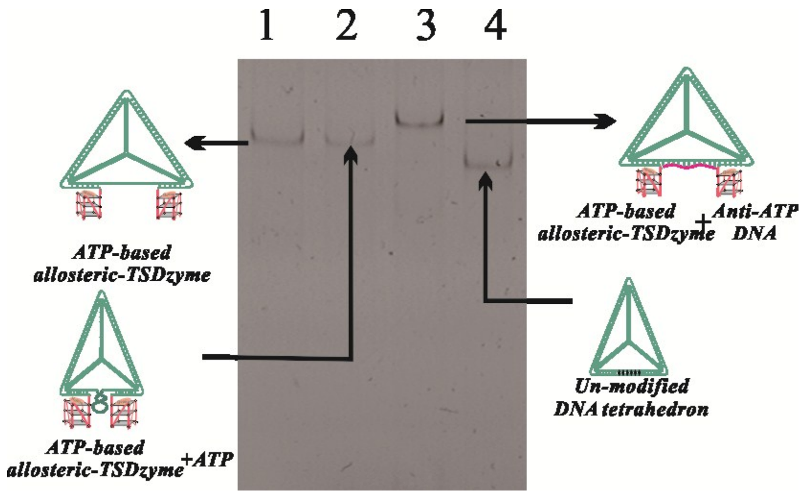

Figure S6. 10% Native gel electrophoresis analysis for the formation of ATP-based allosteric-TSDzyme. Lane 1 stands for allosteric-TSDzyme, Lane 2 stands for allosteric-TSDzyme added with ATP. Due to ATP is a small molecule, there is not any difference between Lane 1 & 2. Lane 3 stands for allosteric-TSDzyme added with atp-DNA, which is the complementary DNA for ATP aptamer. Compared with Lane 1 & 2, the lower mobility of this combination indicates that the ATP aptamer motif in allosteric-TSDzyme is free to bind with ATP and change its configuration. Lane 4 stands for un-modified Tetrahedron as control.

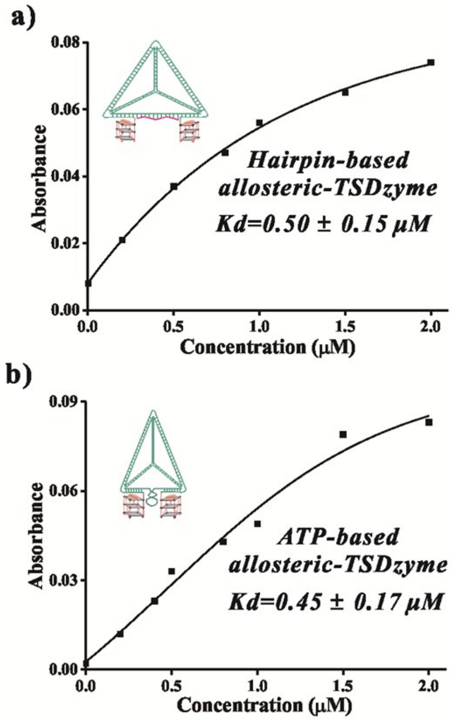

Figure S7. The dissociation constant (K_D) of Allosteric-TSDzymes (hairpin) and (ATP).

Table S2. Free energy of formation of the hemin/G-quadruplex complex in solution (ΔG_{free}) and on the scaffold (ΔG_{tetra}). Standard deviations were averaged from at least three independent experiments.

	$-\Delta G_{free}$ (kJ/mol)	$-\Delta G_{tetra}$ (kJ/mol)	$-(\Delta G_{tetra}-\Delta G_{free})$ (kJ/mol)
TSD **(out)**	32.3±0.6	34.9±0.7	2.6±0.9
TSD **(in)**		35.1±0.5	2.8±0.8
Hairpin-based allosteric-TSD	33.8±0.4	35.9±0.7	2.2±0.9
ATP-based allosteric-TSD		36.2±0.9	2.4±1.0

Table S3. Kinetics and dissociation constants for bi-DNAzyme. Standard deviations were averaged from at least three independent experiments.

	K_M (mM)	K_D (μM)
0 bp	7.84±0.18	4.1±1.0
5 bp	2.22±0.17	1.2±0.2
10 bp	2.15±0.06	1.3±0.2
15 bp	1.93±0.13	1.3±0.4
20 bp	2.16±0.18	1.0±0.2
